# Gender and the Body Size Aftereffect: Implications for Neural Processing

**DOI:** 10.3389/fnins.2019.01100

**Published:** 2019-10-18

**Authors:** Kevin R. Brooks, Evelyn Baldry, Jonathan Mond, Richard J. Stevenson, Deborah Mitchison, Ian D. Stephen

**Affiliations:** ^1^Body Image and Ingestion Group, Department of Psychology, Macquarie University, Sydney, NSW, Australia; ^2^Perception in Action Research Centre, Faculty of Human Sciences, Macquarie University, Sydney, NSW, Australia; ^3^Centre for Rural Health, University of Tasmania, Launceston, TAS, Australia; ^4^Translational Health Research Institute, School of Medicine, Western Sydney University, Sydney, NSW, Australia

**Keywords:** adaptation, aftereffects, body image, body size and shape misperception, gender, neural representation

## Abstract

Prolonged exposure to wide (thin) bodies causes a perceptual aftereffect such that subsequently viewed bodies appear thinner (wider) than they actually are. This phenomenon is known as visual adaptation. We used the adaptation paradigm to examine the gender selectivity of the neural mechanisms encoding body size and shape. Observers adjusted female and male test bodies to appear normal-sized both before and after adaptation to bodies digitally altered to appear heavier or lighter. In Experiment 1, observers adapted simultaneously to bodies of each gender distorted in opposite directions, e.g., thin females and wide males. The direction of resultant aftereffects was contingent on the gender of the test stimulus, such that in this example female test bodies appeared wider while male test bodies appeared thinner. This indicates at least some separation of the neural mechanisms processing body size and shape for the two genders. In Experiment 2, adaptation involved either wide females, thin females, wide males or thin males. Aftereffects were present in all conditions, but were stronger when test and adaptation genders were congruent, suggesting some overlap in the tuning of gender-selective neural mechanisms. Given that visual adaptation has been implicated in real-world examples of body size and shape misperception (e.g., in anorexia nervosa or obesity), these results may have implications for the development of body image therapies based on the adaptation model.

## Introduction

While human perception is impressive in many respects, it is by no means infallible. For example, many humans make consistent errors when estimating the size and shape of their own bodies – a phenomenon known as body size and shape misperception (BSSM; Challinor et al., 2017; Brooks et al., 2019b). It has been shown that some individuals who are obese or overweight may misperceive their body size as being normal (Truesdale and Stevens, 2008; Wetmore and Mokdad, 2012), while some individuals who are underweight, including those with the eating disorder *anorexia nervosa*, are prone to overestimate their body size (Probst et al., 1998; Cornelissen et al., 2017; Molbert et al., 2017). This example of regression to the mean for perceived body size has been referred to as “contraction bias” (Cornelissen et al., 2016).

In recent years, perceptual psychologists have sought a causal explanation for BSSM, suggesting that these phenomena may be real-world examples of perceptual aftereffects (Helmholtz, 1924; Challinor et al., 2017; Brooks et al., 2019b). It is well known that prolonged exposure to a particular sensory stimulus (the “adaptor”) causes the perception of subsequently encountered “test” stimuli to be systematically biased. Often this aftereffect involves the test stimulus taking on perceived qualities that are, in a sense, opposite to the adaptor. For example, exposure to a yellow stimulus can lead to a neutral stimulus appearing blue (Helmholtz, 1924; Hurvich and Jameson, 1957). This process is known as visual adaptation. Alongside color, aftereffects have been demonstrated for other low-level stimulus properties such as motion, orientation and spatial frequency, as well as higher-level properties, such as the identity, race or gender of faces (Clifford and Rhodes, 2005). According to the visual adaptation model of BSSM, extended and repeated exposure to large bodies – those of friends or family members for example – may yield an aftereffect of underestimation, such that an individual’s own body viewed in the mirror may appear smaller than it is. Conversely, repeated viewing of thin bodies, such as those of models in the media may cause a fattening aftereffect, leading individuals to overestimate their size (Brooks et al., 2019b; Challinor et al., 2017).

The effects of adaptation have been characterized as a perceptual recalibration, effected by a change in the response properties of cells activated by the adaptation stimulus (Barlow and Hill, 1963; Mollon, 1974; Ibbotson, 2005; Krekelberg et al., 2006). The adjustment of the relationship between objective stimulus qualities and the frequency of neural impulses means that the same sensory input will result in a different pattern of neural responses before and after adaptation, leading to a change of stimulus appearance. This allows investigators an opportunity to probe the neural mechanisms responsible for the processing and representation of sensory stimuli by assessing the magnitude of aftereffects under different experimental conditions, in particular when the properties of adaptors and test stimuli are manipulated.

**FIGURE 1:**
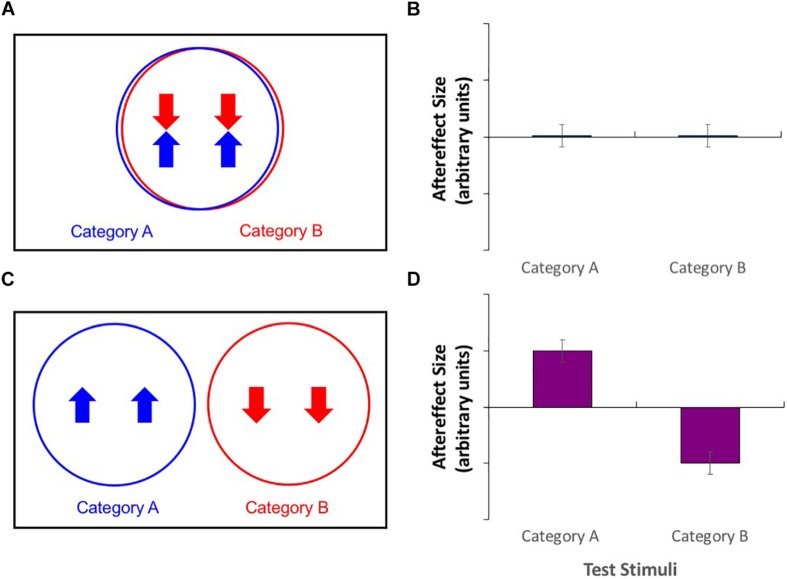
**(A,C)** Venn diagrams of putative neural populations during adaptation to category A stimuli with a high value on a particular stimulus dimension, along with adaptation to category B stimuli with a low value. **(B,D)** Patterns of aftereffects for category A and category B test stimuli. **(A)** Adaptation effects cancel in category agnostic neural populations. **(B)** This produces no measurable aftereffects for either category of test stimulus. **(C)** Opposite direction adaptation effects in category selective neural populations. **(D)** This produces aftereffects whose directions are contingent on the category of the test stimuli.

Take, for example, the paradigm of contingent adaptation. Here, stimuli from two different categories are used for adaptation, each with opposite values on a separate stimulus dimension. For example, this paradigm has been used to investigate the neural substrates underlying the perception of facial structure for different race faces (Jaquet et al., 2008; Gwinn and Brooks, 2013, 2015b). If the two categories (here, faces from different racial groups) are processed by the same neural mechanism (see Figure 1A), then this mechanism should be affected by both the high value of one stimulus category (Caucasian adaptors whose features had been expanded toward the edges of the face) and the low value of the other stimulus category (East Asian adaptors with contracted features), leading to a cancelation of effects and a consequent absence of any measurable aftereffect (Figure 1B). Alternatively, if stimuli from the two categories are processed by separate mechanisms (Figure 1C), then independent contrasting aftereffects should be demonstrated for both stimulus types (Figure 1D). The result was simultaneous aftereffects of contraction for Caucasian test faces and expansion for East Asian test faces, suggesting that faces belonging to these categories are processed separately. Similar results have been shown for faces of different genders (Little et al., 2005; Jaquet et al., 2008; Gwinn and Brooks, 2015a) and even species (Little et al., 2008; Gwinn and Brooks, 2015a).

**FIGURE 2:**
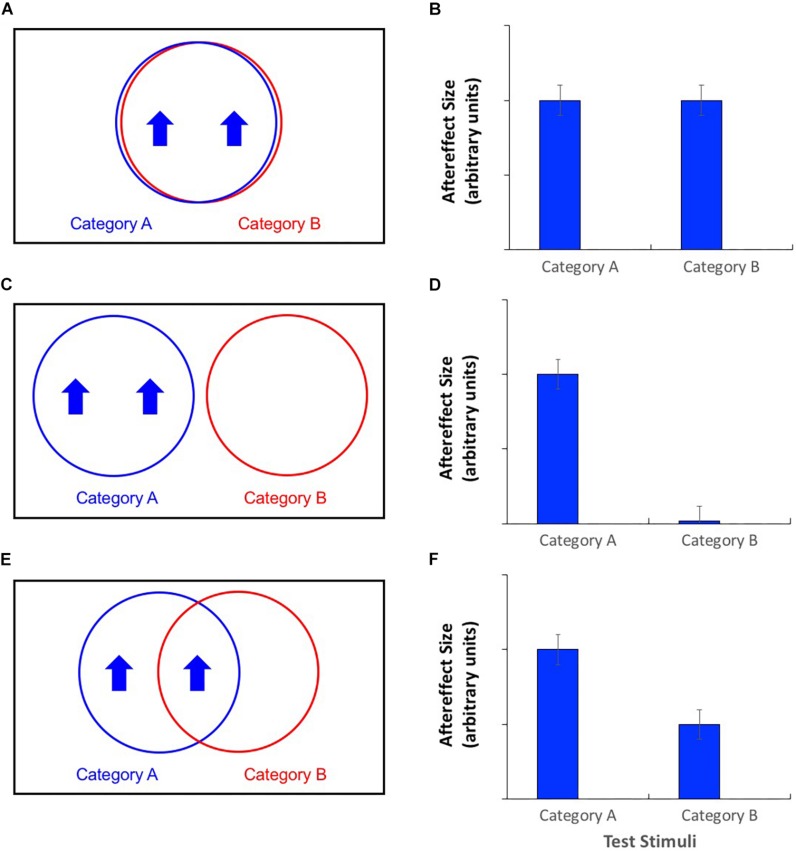
**(A,C,E)** Venn diagrams of putative neural populations during adaptation to category A stimuli with a high value on a particular stimulus dimension. **(B,D,F)** Patterns of aftereffects for category A and category B test stimuli. **(A)** Category agnostic neural populations produce **(B)** complete cross-adaptation, i.e., aftereffects are equal in magnitude regardless of the test stimulus category. **(C)** Category selective neural populations produce **(D)** no cross-adaptation, i.e., aftereffects are non-existent for test stimuli from category B. **(E)** Partially category selective neural populations produce **(F)** partial cross-adaptation, i.e., aftereffects for category B test stimuli are significant, but smaller than for category A test stimuli.

An alternative approach involves the technique of cross-adaptation. Unlike contingent adaptation, in this case an observer is exposed to only one type of adaptation stimulus. Insights into the details of neural representations are gained by assessing changes in the magnitude of aftereffects as the experimenter manipulates the similarity between the adaptor and the test stimulus. When the adaptor and test are highly similar, they will be processed by the same neurons (Figure 2A), and the potential for cross-adaptation will be maximal, such that aftereffects are similar in magnitude regardless of which test stimulus is used (Figure 2B). When adaptors and test stimuli differ, the magnitude of the aftereffect should decline to the extent that the neural populations recruited by the test stimulus are separate from those responsible for processing the adaptor. If entirely separate neural populations process the two categories (Figure 2C), then there should be no cross-adaptation at all, i.e., no recorded aftereffect when adaptor and test differ (Figure 2D). If the neural populations overlap to some extent (Figure 2E), partial cross-adaptation (i.e., a smaller aftereffect magnitude) should result (Figure 2F). Jaquet and Rhodes (2008) reported face aftereffects when adaptors and test stimuli differed in terms of their gender, suggesting an overlap in terms of the neural units responsible for processing these two stimulus categories. However, these cross-adaptation effects were smaller than the “simple-adaptation” effects observed when adaptors and test stimuli belonged to the same gender category, suggesting that this overlap was only partial.

Here, we use these two complementary paradigms to probe the neural mechanisms responsible for the perception of the size and shape of male and female bodies. While experiment 1 employs the technique of contingent adaptation, experiment 2 uses cross-adaptation.

## Experiment 1: Contingent Adaptation

In this experiment, participants are adapted simultaneously to images of male and female bodies that have been manipulated in opposite directions to appear either heavier or lighter than normal. If the perception of body size is mediated by a single neural population regardless of gender, both male and female adaptation stimuli would be expected to affect this mechanism, with their equal-and-opposite aftereffects canceling each other. This pattern of results represents the null hypothesis. As a result, no aftereffect should be observed for stimuli of either gender. However, if there is a degree of functional separation between the neural populations processing body size for males and female bodies, then the opposing adaptors should each affect a separate set of neurons, allowing us to hypothesize aftereffects with a direction that is contingent on the gender of the test stimulus being used.

### Materials and Methods

#### Participants

Twenty-seven Caucasians aged between 18 and 40 participated in Experiment 1 (14 females, 13 males, *M*_age_ = 21.22, *SD* = 0.51). Of these, 15 were undergraduate psychology students and twelve were friends and family of one of the researchers. All participants were naïve as to the hypotheses, had normal or corrected-to-normal vision and gave written informed consent before participation. Both experiments were approved by the Macquarie University Human Research Ethics Committee (MQ HREC Ref 5201829753348, approved 03/05/2018).

#### Design

The experiment used a 2 × (2) mixed factorial design. The between-subjects factor – adaptation condition – had two levels: expanded male/contracted female (Male+/Female−) or contracted male/expanded female (Male−/Female+). The within subjects factor was the gender of the test stimuli with two levels: male and female. We measured the Point of Subjective Normality (PSN): the body size that the participant selected as appearing normal. PSN was measured before adaptation, as a baseline, and again after adaptation, to assess the effects of exposure. The dependent variable for all analyses was ΔPSN, calculated by subtracting pre-adaptation from post-adaptation scores. Positive ΔPSN values indicate that participants had selected larger bodies after adaptation. This suggests that an aftereffect of contraction had occurred, causing observers to compensate for the perceptual size reduction by declaring a larger stimulus to have a normal appearance. Conversely, negative ΔPSN values indicate that participants had selected smaller bodies after adaptation, suggesting that expansion aftereffects had occurred.

#### Stimuli

Body stimuli were created from photographs of males and females that were accessed from an archive of photos held by the Macquarie University Body Image and Ingestion Group. Images were standardized in terms of their viewpoint, pose, clothing, background, lighting and camera settings (see Brierley et al., 2016). In an attempt to minimize noise in the results due to variability in the stimuli selected, images of individuals with similar body compositions were chosen. The 18 body identities whose body fat percentage was closest to the mean of images of their gender in the archive were chosen for females (*M* = 24.53, *SD* = 6.80) and for males (*M* = 15.88, *SD* = 6.98). Within each set, all images were also within one standard deviation of the mean for BMI (females *M* = 21.66, *SD* = 2.96; males *M* = 24.08, *SD* = 4.35) and for muscle percentage (females *M* = 71.64, *SD* = 6.45; males *M* = 79.30, *SD* = 6.62). Two male and two female bodies were selected to serve as practice identities, and were not used in the data collection phases, leaving 16 experimental stimuli of each gender. Amongst these 16, eight were randomly selected to serve as test identities, while the other eight were used for adaptation.

**FIGURE 3:**
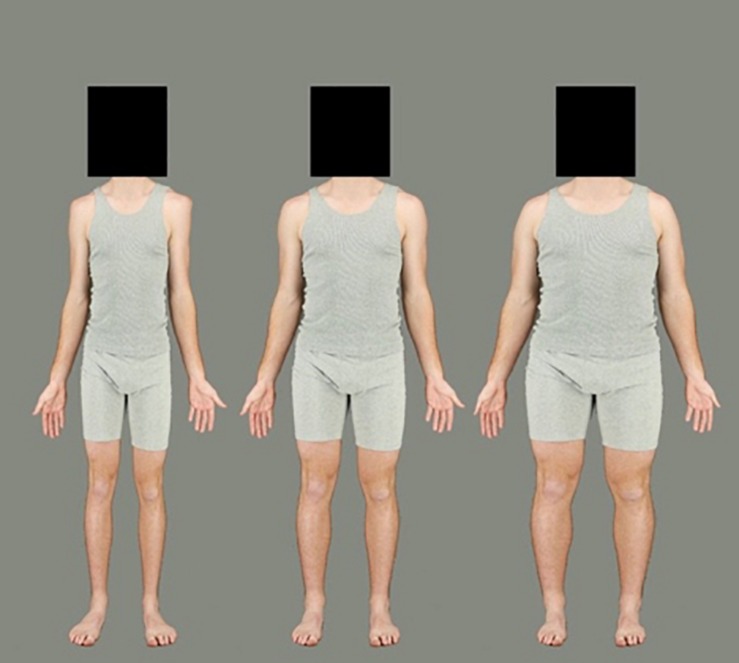
Example of a male identity used in the adaptation phase. The left body represents 50% contraction and the right body represents 50% expansion. The middle is the original image not used in adaptation.

**FIGURE 4:**
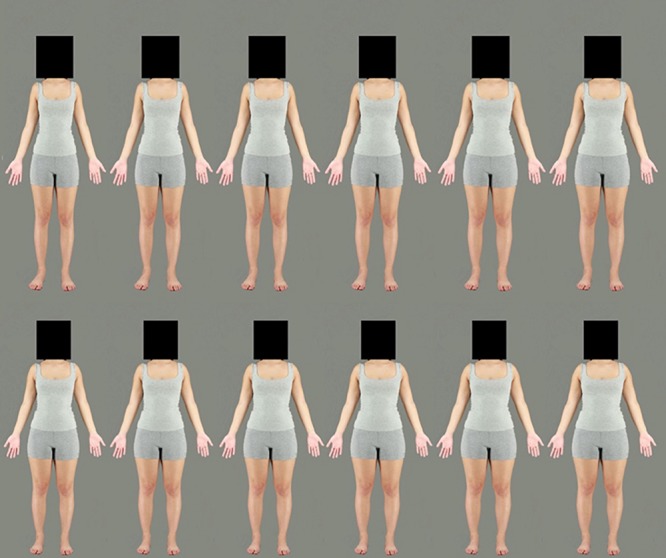
Example of a female identity used in the test phase. Images ranging from the most contracted (–30%) on the top left to the most expanded (+30%) on the bottom right. The original body is not included in this figure.

To simulate variations of body size, Adobe Photoshop CC 2018 was used to create several versions of each of the selected body stimuli. Each photograph was subjected to an identical image manipulation to simulate larger and smaller body sizes using the horizontal “spherize” function. An elliptical region of the image, stretching from neck to ankles, was selected. A feathered edge to this elliptical marquee ensured that the distortion smoothly integrated with the unmanipulated regions of the body (the face/head and feet). To create the two adaptation stimuli, each body stimulus was subjected to a −50% (contraction) and +50% (expansion) horizontal distortion (see Figure 3). For the test stimuli, 13 versions of the images were created in 5% increments from −30 to +30% expansion (see Figure 4). The heads of all stimuli were covered by a standardized black box to eliminate the possibility of face adaptation. All images were set to an aspect ratio of 2:3. Adaptation images were 1094 × 1641 pixels, while test images were presented at 820 × 1230 (3/4 of the size of the adaptation images) to reduce the effects of low-level adaptation (Brooks et al., 2018). Images were presented on a Dell P1130, 21″ color monitor using Matlab version 7, operating Psychophysics Toolbox extensions (Kleiner et al., 2007) and were viewed from a distance of 70 cm.

#### Procedure

In the practice phase, participants were familiarized with the procedure of selecting a normal body size using the body manipulation tool. This allowed participants to adjust the test body to ±30% of its original size by horizontally sliding the mouse from right (expanded image) to left (contracted image). The adjustment involved the display of the 13 test images appearing in sequence, giving the illusion of a smooth transition in body size. When the participant clicked the mouse, the size of the body on the screen was recorded as the PSN value.

In the baseline data collection phase, images of male and female bodies were presented and participants were required to adjust them to the size they perceived as “normal.” On each trial, the initial size of the test body was chosen at random from the 13 possible sizes. There were eight test body identities for each gender and each identity was presented twice. Baseline PSN was calculated separately for male and female test conditions as the average body size selected across the 16 relevant trials.

Adaptation data were collected immediately following the baseline phase. Participants observed a 256 s “initial” adaptation sequence, where they were exposed to alternating images of the eight male and eight female adaptation bodies (e.g., Male + /Female−), each visible for a 2 s duration, repeated eight times. Subsequently, they were required to readjust the eight male and eight female test bodies to a “normal” size. In between each trial there was 12 s of “top up” exposure to maintain levels of visual adaptation before the next test body was presented. Here, a randomly chosen three male and three female adaptors were used. With genders alternating, each was again visible for 2 s. All other details were identical to the baseline phase.

### Results

**FIGURE 5:**
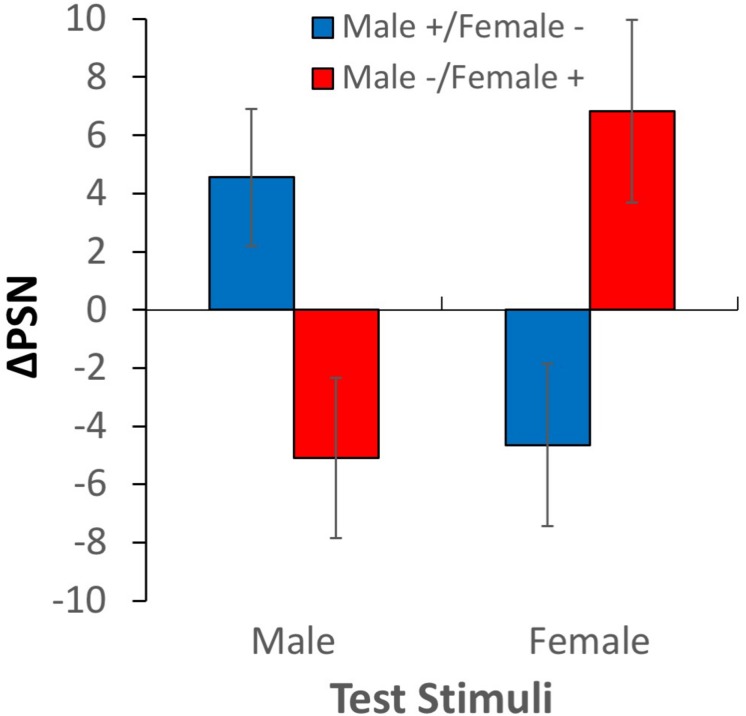
Results of experiment 1. Changes in points of subjective normality for both adaptation conditions and test stimulus genders. Error bars show ± 1 SEM.

The change in the point of subjective normality between baseline and adaptation scores is plotted in Figure 5 for both adaptation conditions, and for male (ΔPSN_male_) and female (ΔPSN_female_) test stimuli. From inspection, it is apparent that adaptation to expanded male bodies and contracted female bodies caused male test bodies to be perceived as smaller and female test bodies as larger (compared to baseline levels). In each case, participants made compensatory adjustments to the test stimuli using the body manipulation tool to reach a positive ΔPSN_male_ and a negative ΔPSN_female_. In contrast, adaptation to contracted male bodies and expanded female bodies caused male test bodies to be perceived as larger and female test bodies to be perceived as smaller than they were before adaptation. These observations were confirmed by a 2-way ANOVA^1^, where a significant interaction between adaptation direction and test gender reaffirmed the presence of contingent aftereffects *F*_(__1__,__25__)_ = 12.045, *p* = 0.002, η_p_^2^ = 0.325. As expected, there was no significant main effect of test gender *F*_(__1__,__25__)_ = 0.201, *p* > 0.05 or adaptation condition *F*_(__1__,__25__)_ = 0.137, *p* > 0.05.

### Discussion

These results are inconsistent with a system with a single, gender-agnostic mechanism for processing the size and shape of body stimuli. Such a system would be exposed simultaneously to expanded and contracted stimuli, resulting in no net adaptation and no measurable aftereffect (see Figures 1A,B). Even if there had been a slight imbalance in the sizes and shapes of male and female stimuli, resulting in an asymmetric adaptation effect in the Male+/Female− compared to the Male−/Female+ adaptation condition, this should have resulted in equivalent PSNs when male and female bodies were used as test stimuli. This was clearly not the case. Instead, there is evidence of opposite aftereffects contingent on the gender of the test stimuli. This is consistent with the existence of gender-selective mechanisms that are engaged when the visual system processes body size and shape.

While it is clear that the systems processing body size and shape for male and female bodies are independent to some degree, the extent of their independence cannot be revealed by this experiment. It is possible that only some of the neurons are gender-selective, resulting in the observed contingent aftereffects. Meanwhile, other cells processing size and shape may be excited by body stimuli regardless of their gender, and in these neurons the opposite adaptation effects would cancel. As the degree of cancelation occurring in experiment 1 cannot be known, a different approach is required to determine whether the perception of body size and shape is mediated by fully or partially gender-selective mechanisms.

## Experiment 2: Simple/Cross Adaptation

In this experiment, observers are adapted to a single set of bodies of a given gender (male or female) that have either been expanded to simulate higher, or contracted to simulate lower body mass. *A priori*, it was considered unlikely that this could result in complete cross-adaptation (i.e., equivalent aftereffects for male and female test stimuli), as the gender-agnostic system that would produce such results has already been ruled out by experiment 1 (Figures 2A,B). This lack of a difference between the magnitudes of aftereffects represents the null hypothesis. However, two other possibilities remain, producing competing hypotheses. If judgments of body size and shape involves independent neural populations that are strictly gender selective (Figure 2C), then aftereffects established with one adaptation stimulus should be seen only when testing with stimuli of the same gender, with no transfer of this aftereffect to other-gender test stimuli (Figure 2D). However, if the systems underlying body size and shape perception for male and female stimuli are partially gender-selective (Figure 2E), we should observe a degree of transfer. This means that aftereffects should be significant when adaptation and test stimuli differ in terms of gender, yet these effects should be smaller in magnitude than when they have the same gender (Figure 2F).

### Materials and Methods

This investigation was identical to Experiment 1, except in the following respects. Experiment 2 employed a 2 × 2 × (2) mixed factorial design. The sole within-subjects factor was the gender of the test bodies, with two levels: male and female. The first between-subjects factor – adaptation gender – had two levels: male or female, and second between-subjects factor – adaptation direction – also had two levels: expanded or contracted. Eighty-four Caucasian participants aged between 18 and 40 years old participated in (*M*_age_ = 20.76, *SD* = 3.30). Of these, 67 were undergraduate psychology students and seventeen were friends and family of the researcher. The results of four participants were removed for lack of compliance with instructions, leaving a total of eighty participants: 20 in each condition. Participants observed a 128 s adaptation sequence where they were randomly exposed to stimuli from one of the four adaptation conditions (eight identities, 2 s duration for each, eight repetitions). Between each trial there was a 6 s “top up” consisting of three of the adaptation identities, selected at random, to ensure maintenance of adaptation levels. These durations (initial and top up) match the exposure times for adaptors of each gender used in experiment 1 (i.e., half of the experiment 1 total adaptation duration).

### Results

**FIGURE 6:**
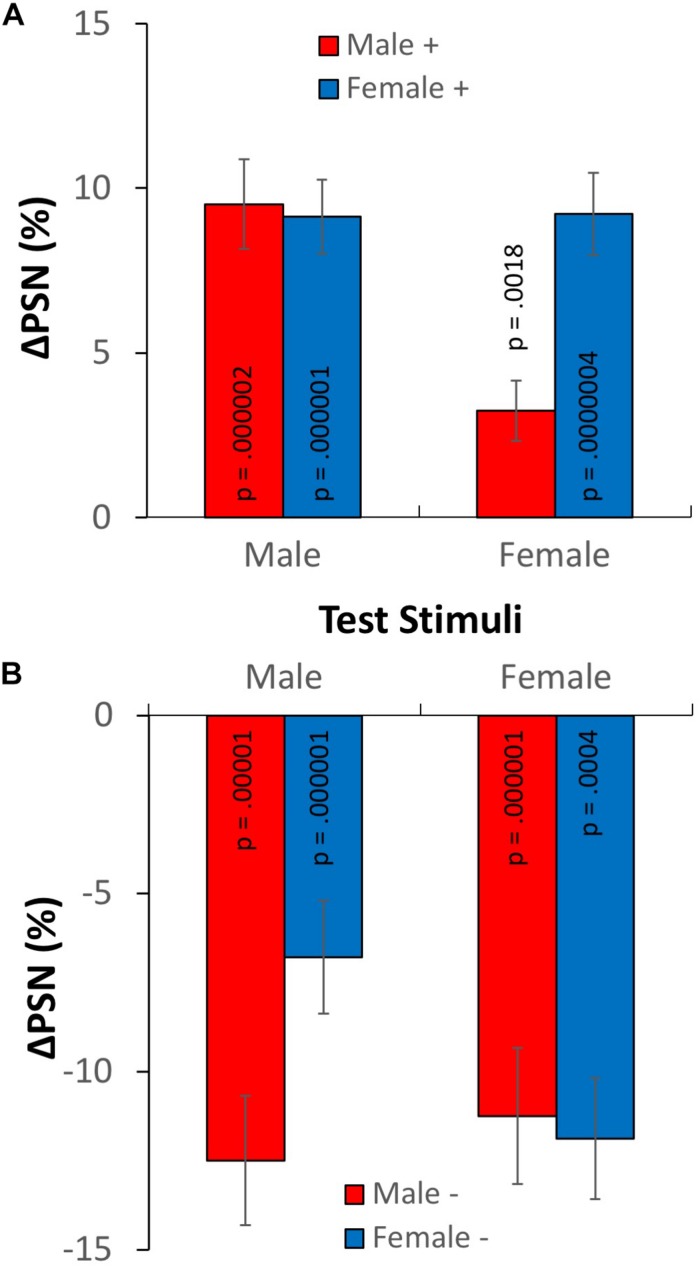
Results of experiment 2. Changes in points of subjective normality for both adaptation conditions and test stimulus genders. **(A)** Adaptation to expanded stimuli. **(B)** Adaptation to contracted stimuli. Error bars show ± 1 SEM.

The results of Experiment 2, in terms of the adaptation-induced change in the point of subjective normality are plotted in Figure 6, for both male (ΔPSN_male_) and female test stimuli (ΔPSN_female_). From informal inspection, it appears that exposure to expanded adaptors causes PSNs to increase (Figure 6A), while exposure to contracted adaptors causes them to decrease (Figure 6B), as expected. As in experiment 1, this is consistent with a contraction aftereffect after adaptation to expanded figures, and an expansion aftereffect following adaptation to contracted figures. When the gender of adaptation and test stimuli match (simple adaptation conditions), these aftereffects are relatively large, on average exceeding a distortion level of 10% (or one fifth of the level of the adapting stimulus). Most importantly, when the gender of adaptation and test stimuli are different (cross-adaptation conditions), aftereffects are present for each adaptation condition, but in each case they are smaller than when adaptor and test have the same gender (simple adaptation).

Formal statistical tests confirmed these preliminary observations. One sample *t*-tests^2^ showed statistically significant adaptation effects for all simple adaptation conditions (smallest effect: *t*_19_ = −6.87, *p* = 0.0004, *d* = 1.54), and all cross-adaptation conditions (smallest effect: *t*_19_ = 3.57, *p* = 0.0018, *d* = 0.80).^3^ A 2 × 2 × (2) ANOVA^1^ showed a significant main effect of adaptation direction *F*_(__1__,__76__)_ = 202.967, *p* < 0.0005, η_p_^2^ = 0.728, confirming the expected difference between the aftereffects in contracted and expanded conditions. In addition, a significant three-way interaction between adaptation direction, adaptation gender and test gender was revealed *F*_(__1__,__76__)_ = 17.758, *p* < 0.0005, η_p_^2^ = 0.189, confirming the predicted difference between simple adaptation and cross-adaptation ΔPSNs.^4^

To further examine the three-way interaction, paired *t*-tests^1^ examined differences between ΔPSN_male_ and ΔPSN_female_ for each adaptation condition. In two cases, simple adaptation effects were significantly larger than cross-adaptation effects. For expanded male adaptors, ΔPSN_male_ values were significantly larger than ΔPSN_female_ values *t*_19_ = 5.201, *p* < 0.0005, *d* = 1.163. Similarly, for contracted female adaptors, ΔPSN_female_ values were significantly larger than ΔPSN_male_ values *t*_19_ = 2.577, *p* = 0.018, *d* = 0.576. However, in the other two conditions, although simple adaptation ΔPSN values were larger than those for cross-adaptation, these differences were not significant (*p* > 0.05).

**FIGURE 7:**
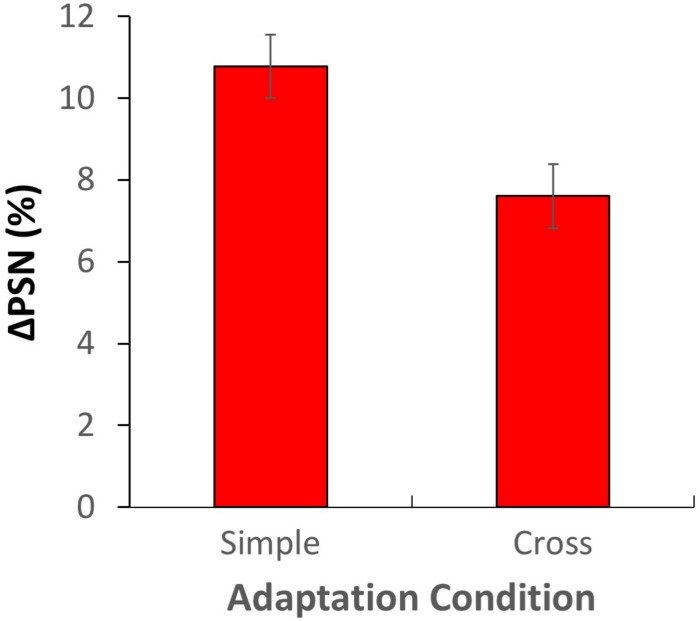
Results of experiment 2, aggregated and recoded in terms of simple and cross-adaptation conditions. Error bars show ± 1 SEM.

An additional analysis was conducted to explicitly examine the overall difference between simple and cross-adaptation ΔPSNs across all adaptation conditions. Here, for each participant, ΔPSN_male_ and ΔPSN_female_ values were recoded as ΔPSN_simple_ and ΔPSN_cross_ (depending on the adaptation gender), and values from contracted adaptation conditions were “rectified” by multiplying by −1 to allow combination with data from expanded adaptation conditions (see Figure 7). Clear aftereffects were observed for both simple adaptation (one-sample *t*-test: *t*_79_ = 13.893, *p* = 6.52 × 10^23^, *d* = 1.553) and cross-adaptation conditions (one-sample *t*-test: *t*_79_ = 9.745, *p* = 1.35 × 10^24^, *d* = 1.089), with significantly larger effects for simple adaptation (*t*_79_ = 3.999, *p* = 0.00014, *d* = 0.447). The cross-adaptation effect was 71% the size of the simple adaptation effect.

### Discussion

Despite the overall demonstration of partial cross-adaptation when conditions are combined, the results of individual conditions are somewhat asymmetrical. While partial adaptation was clear and significant when adaptors were expanded males or contracted females, this effect was not significant when adaptors were expanded females or contracted males. A possible explanation may lie in the gender typicality of certain body shapes. It may be that, in general, stimuli that are expanded to a broader body shape size are viewed as more masculine, while those contracted to a more slender figure may be perceived as more feminine. Hence, broad female adaptors may cause some residual activation (and hence adaptation) of male-selective neurons, leading to a greater than expected degree of cross adaptation, and an inability to demonstrate a significant difference between simple and cross adaptation conditions. Conversely, thin male bodies may cause some unintended adaptation of female-selective neurons, reducing the chances of finding significant differences in this case. While this explanation is promising, it should be remembered that in Experiment 1, the condition combining these adaptors (Male−/Female+) was successful in demonstrating a contingent aftereffect (see Figure 5). It is possible that the direct contrast between the two sequentially presented adaptors accentuated their gender typicality in experiment 1 enough to allow the measurement of different effects for male and female test stimuli. Alternatively, the lack of significance in experiment 2 may simply be the result of noise.

The general demonstration of partial cross-adaptation suggests that the neural populations underlying the processing of size and shape are partially gender-selective (as in Figures 2E,F). Some neurons are activated by stimuli of either gender, and it is these units that are responsible for the transfer of aftereffects between adaptation stimuli from one category and test stimuli from another. However, transfer of the aftereffect is not complete, suggesting that some neurons process stimuli for one gender only, such that their adaptation does not result in an aftereffect when opposite gender test stimuli are used.

## General Discussion

This study is the first to investigate gender-selectivity in the neural populations processing body size and shape. Inferences concerning the properties of these groups of cells are made possible through the psychophysical method of visual adaptation, and the examination of aftereffects while manipulating the congruence between the gender of adaptors and test stimuli. The two experiments provide consistent and complementary findings. First, experiment 1’s contingent aftereffects established a degree of gender independence in the processing of body stimuli. Experiment 2’s observation of partial cross-adaptation confirmed this finding while establishing that the independence is far from complete. Taken together, these findings suggest that a proportion of the underlying neural populations behave in a gender agnostic manner to some extent. Although it is not possible to precisely quantify the level, experiment 2’s observation of approximately 70% cross-adaptation suggests a small-to-medium degree of gender-selectivity. In broad agreement with this, it is notable that in experiment 1, contingent aftereffects were approximately half the size of simple adaptation effects shown in experiment 2, which used the same adaptation stimuli and exposure durations. This is consistent with the idea that in experiment 1, gender-agnostic cells were adapted to bodies of each gender manipulated in opposite directions, and that this led to a degree of cancelation of the adaptation and hence a smaller measured aftereffect.

Although efforts were made to ensure that all details of our body stimuli were standardized, we acknowledge that gender of the subject is not the only difference between the male and female stimulus sets. An reviewer noticed that the neckline of the singlets and the shade of the shorts differ subtly in our male and female stimuli, and hence there exists the technical possibility that if participants encoded these differences, this may constitute a basis for the phenomena of contingent adaptation and partial cross adaptation seen in our study. However, we consider this unlikely. From observation, we find that gender, communicated through body shape, is the most salient difference between the two stimulus sets, and hence the most likely basis for the categorical distinction. While it seems plausible that, either through evolution or through plasticity and perceptual learning, the brain may have developed neural mechanisms that are selective for the natural categories of stimulus gender, it would seem surprising if it contained similar mechanisms selective for the neck-line or shade of shorts used in our study.

The paradigms of contingent and cross-adaptation have been used previously to investigate the properties of neurons processing body size and shape. For example, a recent study showed substantial cross-adaptation between body stimuli that were oriented either at the same angle, or at right angles to the adapting stimulus (Brooks et al., 2018). Substantial cross-adaptation suggested that the judgment of body size and shape is a high-level process, relying on an object-centered frame of reference, rather than a low-level retinotopic one. In addition, Brooks et al. (2016) conducted an investigation similar to the current study, using body stimuli depicting the observer (“self”), and depicting another individual (“other”) matched in terms of gender, race, age and BMI. Contingent adaptation was evident, suggesting a degree of independence in the processing of these two stimulus categories. As in the current study, cross-adaptation was also demonstrated, suggesting that the selectivity for self/other was only partial. In contrast, Gould-Fensom et al. (2019) were unable to show any contingent body size and shape adaptation using bodies from different racial groups (East Asian and Caucasian). A demonstration of 100% cross-adaptation between bodies from the two groups in a second experiment confirmed that the neurons are race-agnostic, suggesting that the lack of contingent aftereffects is likely to be due to substantial transfer and cancelation of aftereffects that are opposite in direction.

The current study presents strong evidence for the existence of partially gender selective mechanisms for the perception of body size and shape, yet there are several matters of interest on which it is unable to shed light. One such issue is the location of the neural structures responsible for our results. Various body-sensitive brain areas have been identified, some of which show modulation of activity when observers view bodies of different sizes. These areas, including the fusiform body area (FBA) and the extrastriate body area (EBA) (Hodzic et al., 2009; Aleong and Paus, 2010; Hummel et al., 2013; Gandolfo and Downing, 2019); the lateral occipital cortex (LOC), and the middle frontal gyrus (MFG) (Mohr et al., 2011) may be considered candidates for the neural locus of body size and shape judgments. More recent fMRI work has revealed at that both gender and weight can be decoded from distributed body-selective areas (including EBA and FBA) even when the classifier is trained and tested with body stimuli of different heights (Foster et al., 2019). However, this study is unable to reveal whether sex-selective cells in these regions also process body size (i.e., weight), as suggested by our contingent and cross-adaptation results, or whether these two stimulus attributes are processed by separate cells in the same general brain vicinity (for example, within an fMRI voxel). Body size and shape judgments may depend on responses in various neural populations in different anatomical locations or at different levels of the processing chain, and each of these may show different degrees of gender selectivity. For example, it is possible that at one level, cells are strictly gender specific (responding only to the gender for which they are selective, as in Figure 2C), but at another level, cells are completely gender-agnostic (responding equally to all bodies regardless of their gender, as in Figure 2A). While the contingent aftereffects of experiment 1 would be explained by responses in the former pool of neurons, the cross-adaptation demonstrated in experiment 2 would be explained by activity in the latter.

Although the Venn diagrams in Figures 1, 2 offer an intuitive depiction of size and shape perception mechanisms with varying degrees of gender selectivity, it should be acknowledged that they are somewhat simplistic. For example, these diagrams show a maximum of three types of cell: those that are strictly male-selective, those that are strictly female-selective, and those that are gender agnostic, being equally well stimulated by male or female body stimuli. Given that neither gender nor sex are strictly dichotomous variables, and that observers perceive bodies as varying in gender typicality (Palumbo et al., 2013), this is likely to be an oversimplification, and it may be more appropriate to consider the responses of cells to body stimuli along a continuum of gender typicality. Although the gender tuning of body selective cells has not been measured, based on the encoding of gender for faces (Gwinn and Brooks, 2015a), it seems likely that cells may show broad tuning to the gender of stimulus bodies, with vigorous responding to figures that are typical of the gender to which the cells are tuned, and a gradual reduction of activity as the gender typicality of the stimulus is reduced.

While neuroscientists interpret the results of high-level adaptation experiments in terms of the recalibration of response properties amongst various neural populations (Barlow and Hill, 1963; Krekelberg et al., 2006), psychologists – particularly when discussing the effects of face adaptation – tend to couch these effects in terms of the malleability of perceptual “norms” (Clifford and Rhodes, 2005; Jaquet and Rhodes, 2008; Jaquet et al., 2008). Perceptual norms are averages of all stimuli of a particular category that an individual has encountered. For example, an observer’s face norm would have average features located in the mean position across all faces that the observer has seen. Every time a new face is encountered, it is encoded in terms of its deviation from the norm face along a number of image dimensions in “face space” (Valentine, 1991). The effect of adaptation is to shift the norm toward the adapting stimulus, such that subsequently seen stimuli tend to be perceived to be, in this space, opposite to the adaptor (Clifford and Rhodes, 2005). When contingent adaptation is demonstrated, for example between categories such as male and female faces, this is seen as evidence for distinct norms for each of the categories, each of which has been biased in opposite directions (Jaquet and Rhodes, 2008; Jaquet et al., 2008; Gwinn and Brooks, 2013, 2015a,b). In terms of bodies, a similar interpretation can be applied to our findings, using a “body space” framework (Rhodes et al., 2013; Sturman et al., 2017). While the demonstration of contingent adaptation can only be accounted for by separate norms, cross-adaptation suggests that a gender-agnostic norm also exists, possibly at another level of processing.

The current study is not the first to examine body adaptation in the context of gender. A recent study by Brooks et al. (2019a) showed that aftereffects of perceived muscle and fat levels are larger in magnitude when the stimuli (adaptation and test) match the gender of the observer.^5^ Although the current study focuses on inferences regarding the neural processing of body stimuli, it may also carry implications for real-world body image issues such as BSSM, especially when combined with the aforementioned results. As mentioned in the introduction, it has recently been suggested that perceptual aftereffects may underlie these misperceptions (Brooks et al., 2019b; Challinor et al., 2017). If this is true, then the current results offer predictions regarding the manifestation of BSSM. If extensive exposure to certain body types in the media or in one’s local environment does cause aftereffects to be experienced when viewing one’s own body in the mirror, it would be expected that the gender of the bodies being viewed would have an influence. For example, whereas a male who extensively views female fashion models on TV or social media may experience a degree of BSSM (overestimation), a larger effect would be expected for females consuming the same media. Similarly, if an overweight family includes many more males than females, we may expect BSSM to develop in the form of underestimation. However, males would be expected to suffer to a greater degree in this case. While it cannot be asserted that visual adaptation is the underlying cause of BSSM, the use of adaptation and the observation of aftereffects serves as a potent tool to examine the neural and psychological underpinnings of body size and shape misperception. This is an essential first step in explaining, and, perhaps, informing therapeutic interventions for, conditions involving body size and shape misperception (BSSM).

## Data Availability Statement

The datasets generated for this study are available on request to the corresponding author.

## Ethics Statement

Both experiments were reviewed and approved by Macquarie University Human Research Ethics Committee. All participants provided informed consent in writing.

## Author Contributions

KB was responsible for the conception and design, the analysis and interpretation of data, original drafting, and final approval of the submitted version. EB was responsible for aspects of the design, the analysis and interpretation of data, aspects of original drafting, and final approval of the submitted version. JM, RS, DM, and IS made substantial contributions to the interpretation of data, critical revisions for important intellectual content, and final approval of the submitted version.

## Conflict of Interest

The authors declare that the research was conducted in the absence of any commercial or financial relationships that could be construed as a potential conflict of interest.

## Footnotes

1 Conducted using SPSS v.25.

2 Performed using Microsoft Excel 2016.

3 These would be considered significant effects even with a conservative Bonferroni-corrected family wise critical *p*-value of 0.00625 (eight pairwise comparisons).

4 In addition, there were significant main effects of adaptation gender *F*_(1,76)_ = 4.287, *p* = 0.042, ηp^2^ = 0.053 and test gender *F*_(1,76)_ = 11.10, *p* = 0.001, ηp^2^ = 0.127. These main effects held no theoretical interest and hence were not investigated further.

5 In addition, Palumbo et al. (2013) demonstrated that exposure to typical male or female bodies could cause a shift in the perceived masculinity/femininity of otherwise androgynous-looking bodies. As in Brooks et al. (2019b) effects were largest when adaptation stimuli matched the gender of the observer.
